# Widely conserved miRNAs in buffalo milk extracellular vesicles survive gastrointestinal digestion and potentially target neural and immunomodulatory contexts

**DOI:** 10.3389/fnut.2025.1685349

**Published:** 2025-10-16

**Authors:** Olubukunmi Amos Ilori, Jessie Santoro, Diana Marisol Abrego-Guandique, Paola Tucci, Giovanni Smaldone, Erika Cione

**Affiliations:** ^1^Department of Pharmacy, Health and Nutritional Sciences, University of Calabria, Rende, Italy; ^2^IRCCS SYNLAB SDN, Naples, Italy; ^3^Department of Health Sciences, University of Magna Graecia Catanzaro, Catanzaro, Italy

**Keywords:** miRNA, extracellular vesicles, buffalo milk, functional analysis, immune modulation

## Abstract

MicroRNAs (miRNAs) are small non-coding RNAs with unique functions. Their presence in human milk raises the possibility of accumulation along the food chain. Buffalo milk extracellular vesicles, as other milks, are a known source of dietary miRNAs. However, information on the digestive stability of miRNAs remains limited, which is a prerequisite for understanding their *in vivo* functionalities. To this, the presence of widely conserved miRNAs: miR-10a-5p, miR-24-3p, miR-25-3p, miR-26a-5p, miR-27b-5p, miR-33a-5p, miR-103a-3p, miR-125b-5p, miR-130a-3p, miR-133a-3p, miR-138-5p, miR-139-5p, miR-141-3p, miR-148a-3p, miR-153-3p, miR-199a-3p and miR-223-3p, were assessed in isolated extracellular vesicles, extracted from buffalo milk. The miR-10a-5p, miR-24-3p, and miR-130a-3p, were not detected in raw buffalo milk. Therefore, we simulated the gastrointestinal digestion using INFOGEST 2.0 and extracted extracellular vesicles from the digest. Apart from particle numerosity, which differed significantly, from 1.2 × 10^11^ ± 5.3 × 10^9^ particles/mL in raw milk to 9.53 × 10^10^ ± 1.2 × 10^9^ particles/mL in digested milk, the extracted extracellular vesicles showed no structural differences before and after digestion. The miRNA cargo exhibited a similar pattern, except miR-141-3p, miR-153-3p, both increased slightly, and miR-223-3p, which increased substantially; miR-148a-3p, which decreased; and miR-33a-3p, which was no longer detectable after digestion. The bioinformatics analysis of the overall 13 miRNAs detected post-digestion, concertedly target neural and immunological contexts, with an MHC-mediated antigen processing and presentation. The prospect offered highlights the potential of milk, through its EV-miRNA fraction, to impact inflammatory responses in the neurodevelopmental processes of the benefiting offspring, and by extension, dairy consumers. However, relevant *in vitro* and *in vivo* investigations are needed to demonstrate the post-digestion transfer of these nucleic acids from the concerned dietary sources and their effect on target tissues.

## 1 Introduction

The primary relevance of effector systems employing small RNAs in the formation of RNA-based interference (RNAi) systems might have been the formulation of a defense structure against extraneous nucleic acid molecules (1). However, this structure has diversified in such a way that endogenously produced RNA molecules are recruited to regulate gene expression. Hence, in multicellular contexts, these systems are composed of effector proteins that mediate the silencing of nucleic acids, such as RNA-Induced Silencing Complexes (RISC), which are responsible for sequence recognition relevant to the system's functionality, and nucleases that process the nucleic acids (2). A major, well-documented nucleic acid component of RNAi is the microRNA (miRNA), which is distinguished from other small RNAs by its highly precise excision from imperfect stem-loop structures residing in the primary miRNA transcripts (3). It is so named due to its short length, typically ranging from 18 to 25 nucleotides, which is approximately the length of a standard PCR primer. miRNAs pair with the 3′ UTR of target mRNA transcripts to repress their translation or cause their cleavage; hence, they are generally regarded as repressors of gene expression.

Due to their vital roles across diverse biological contexts, miRNAs are considered an essential feature of plant and animal development (4, 5). This indicates the plausibility of their ubiquity in any products that can be considered food. To this, the first food of humans is breast milk, in which miRNAs were identified (6). It was borne out of the need to explore other possible vertical transfer of genetic material besides sexual reproduction, but has since then gone on to become a basis of food-related miRNA studies. It is therefore not surprising that milk is the most documented in terms of miRNA make-up. A basic functionality reserved for milk-related miRNAs is the regulation of immune processes in the receiving offspring (6–8). However, concerning their contribution to literature, the identification of miRNAs in food has been vital in exploring the concept of cross-kingdom (9) and cross-species miRNA transference (10).

Notwithstanding, the varying contributions of milk miRNAs to overall biological function have been elaborated upon over the years. This ranges from embryogenesis (11), angiogenesis (contributing to wound-healing) (12), epithelial-mesenchymal transformation-related changes (13), regulation of adipogenesis (14), fibrogenetic potential in liver cirrhosis (15) to the modulation of apoptotic rate, stress, invasion, migration, and clonogenicity of cancer cells (16, 17). Several of these processes are regulated through the control of inflammatory or immune-related pathways. One crucial factor that may preserve the function of dietary miRNAs is their encapsulation in protective structures. These lipid bilayer vesicles are the smaller form of extracellular vesicles (EVs), ranging from 30 to 200 nm, formed by the invagination of endosomes from the mammary gland cell membrane (18) as a part of cell-to-cell communication, taken up through endocytosis by recipient cells which may be routed to the endoplasmic reticulum (ER) and then, lysosomes, for cargo release and mediation of gene expression (19). They confer a substantial (ten-fold) level of protection on the contained miRNAs against conditions that may impede their delivery *in vivo*, especially gastrointestinal digestion (20–22). With the consumption of commercial dairy milk being a global norm, milk EVs are crucial in the interspecies delivery of miRNAs and the consequential regulation of target genes (23).

The conservation of miRNAs across species makes the discussion of cross-species miRNA transfer even more relevant. Particularly in the animal phyla, where mismatches between the relative target and miRNA sequence are more tolerated (24), miRNAs from a species can pair with a target from another species. What's more, the targets of these miRNAs are equally conserved (25), although variation exists in the sites and timing of expression of the miRNAs (26). Within vertebrates, there are more similarities in the sequence of these miRNAs (27), suggesting the possibility that foods offered by animals provide miRNA homologs of high complementarity (28).

Buffalo milk (BuM) accounts for a significant portion of the global dairy output. Like other milks in the dairy sector, it has been garnering attention regarding certain components that offer benefits beyond the classical nutritional outlook. Exosomes from BuM contain and can be a biologically efficient shuttler of bovine miRNAs, especially immune-related ones, which were found to be higher than in cow milk (16, 29). These miRNAs were documented to be protected from the stress that could result from household handling conditions (30), suggesting that selected exosomal BuM miRNAs may serve as biomarkers of milk quality. Despite the complexity of dairy milk being affirmed and the biological efficacy of some of the components being documented, comparatively little is known regarding the feasibility of these functions *in vivo*. A step toward this understanding is examining the possibility of these fractions surviving gastrointestinal digestion. Hence, this study aimed to investigate the stability under simulated gastrointestinal conditions of buffalo milk extracellular vesicles (BuM-EVs) as well as their highly conserved miRNA cargo with the highest alignment statistics with the *homo sapiens* homologs.

## 2 Materials and methods

### 2.1 Sample collection

Raw buffalo milk samples were obtained from pools of milk obtained as part of the routine milking procedure from a dairy farm (Il Caseificio Polito, Agropoli SA, Italy) into sterile urine containers. As a result, no ethical approval was sought for this study. The sample was transported (in 24 h) to the laboratory, where it was aliquoted into sterile Falcon tubes and subjected to subsequent treatments and analyses or stored at −80°C.

### 2.2 *In vitro* gastrointestinal digestion

All reagents and enzymes used, unless otherwise stated, were supplied by Sigma-Aldrich (Schnelldorf, Germany). The gastrointestinal digestion of the milk samples was simulated according to the INFOGEST 2.0 (31). Prior enzyme assays were done to determine the activity and concentration of porcine pepsin (Sigma-Aldrich, Germany), leporine pepsin and gastric lipase (Lipolytech, Marseille, France), porcine pancreatic trypsin (Sigma-Aldrich, Germany), and bovine bile (Sigma-Aldrich, Germany). The human salivary α-amylase (Sigma-Aldrich, Germany) used was not assayed; hence, the supplier-indicated activity was adapted. BuM (20 mL) was treated to oral digestion by mixing with a simulated salivary fluid containing human salivary α-amylase (75 U/mL) and incubating in an agitating incubator (711/CT+ VDLR Mixer, ASA srl, Milan, Italy) for 2 min. This was followed by the gastric phase, where the simulated gastric fluid (pH 3), containing porcine pepsin and rabbit gastric extract solutions, was added to obtain respective final pepsin and gastric lipase activities of 2,000 U/mL and 60 U/mL. The mixture was incubated for 120 min, and the result of the incubation was subjected to intestinal digestion treatment with simulated intestinal fluid (pH 7) containing sonicated porcine pancreatin suspension (100 U trypsin/mL) (32) and bovine bile solution (10 mM) and similarly incubated. Subsequently, the activity of the digestive enzymes was halted by heat shock (MOD. 1800-D Thermostatic Bath, F.lli Galli, Milan, Italy) at 85°C for 15 min, before separation of the soluble/‘digestible' fraction by centrifugation (4,470 × g for 15 min). This was stored at −20°C until further analysis.

### 2.3 Extracellular vesicle isolation

Raw and digested BuM samples were immediately processed upon collection. All samples were centrifuged first at 2,000 × g to remove milk cells, debris, and milk fat globules. Next, the pellet was discarded, and the supernatant was used for another centrifugation step at 10,000 × g. Milk supernatants were filtered twice using a 0.45 μm syringe filter (GVS North America, Sanford, USA) and processed for EVs separation using differential ultracentrifugation (DUC). Specifically, milk samples were centrifuged first at 30,000 × g for 1 h using the MLA-50 Fixed-Angle Rotor and OPTIMA MAX-XP (Cat# 393315, Beckman Coulter, USA). This step was necessary to remove larger EVs and milk contaminants. Therefore, another filtration step was performed on the milk sample prior to obtaining EV pellets. Moreover, milk supernatants were subsequently processed for two rounds of ultracentrifugation at 200,000 × g for 90 min at 4 °C to obtain the EV pellet, which was washed with 0.22 μm filtered PBS (Cat# 14190, Gibco) by ultracentrifugation. Finally, enriched EV pellets were resuspended in 500 μL of 0.22 μm filtered PBS and used for further analysis.

### 2.4 Extracellular vesicle characterization

#### 2.4.1 Dynamic light scattering

Zeta potential (ZP) of BuM-EVs was analyzed by dynamic light scattering (DLS) with Zetasizer Nano ZS 374 (Malvern). The ZP of the EV isolates was measured three times at 25 °C under the following settings: angle of detection backscatter, 3 repeats per measurement, and an equilibration time of 60 s, while analyzing the data with the ZetaView software.

#### 2.4.2 Nanoparticle tracking analysis

Particle concentration and size of BuM-EVs were analyzed using Nanoparticle Tracking Analysis (NTA) (NanoSight NS300, Malvern Instruments Ltd, Malvern, UK). EV samples were diluted 1:100 and automatically injected into the NTA system under constant flow conditions (flow rate = 50). The detection threshold during analysis was selected to ensure that only distinct nano-objects were analyzed and any artifacts were removed. Five × 60-s videos of the particles in motion were recorded and analyzed using NTA 3.2 software.

#### 2.4.3 Scanning electron microscopy

The morphology of BuM-EVs was assessed using scanning electron microscopy. As previously described (33). All EV samples were suspended in 2% paraformaldehyde (PFA) and incubated at 4 °C overnight. Subsequently, samples were ultracentrifuged at 100,000 × g for 2 h, and the fixed EV pellets were resuspended in 200 μL of deionised water. Ten (10) μL of fixed samples were deposited on metallic stubs and coated with gold before imaging. Finally, EV samples were examined using the PHENOM PROX scanning electron microscope (SEM).

### 2.5 Total RNA extraction

Total RNA isolation from the raw and digested BuM-EVs was performed using the bead-binding technology of the KingFisher Duo Prime Magnetic Particle Processor (Thermo Fisher Scientific, Waltham, MA, USA) with MagMAX mirVana Total RNA Isolation Kit (Thermo Fisher Scientific, Waltham, MA, USA). A 100 μL of the EV isolate was added to the wells in the second row of a 96-Deep-Well Plate. To monitor total RNA extraction efficiency, 3 μL 1 pM cel-miR-39-3p was added to the samples before extraction. Extraction, irrespective of the extraction batch, showed consistent recovery with a per cent Ct variability of 3.3%. Extraction was performed in duplicates. Subsequent steps of the extraction procedure followed the kit's accompanying instructions for high-throughput RNA isolation from serum and plasma samples. RNA concentration of the extract was quantified using a Thermo Scientific NanoDrop One instrument (Thermo Fisher Scientific, Waltham, MA, USA).

### 2.6 Quantitative reverse transcription PCR

The 17 miRNAs examined in this study were selected based on widely conserved miRNAs annotated in miRBase (https://www.mirbase.org/) with 100% alignment with the corresponding human homologs (Supplementary Table S1) (34). Reagents and kits used in the qRT-PCR were procured from Life Technologies Europe (Milan, Italy). Total RNA containing 2 ng was used in a Poly(A) reaction with the Taqman Advanced cDNA Synthesis kit. The reaction product was funneled into adaptor ligation, reverse transcription and cDNA synthesis reactions as indicated by the manufacturer. The PCR reaction was set up with 5 μL of the diluted cDNA (1:10 with RNAse-free water). The probes used in this study are presented in Supplementary Table S2, in compliance with the Minimum Information for the Publication of Real-Time Quantitative PCR Experiments (MIQE) guidelines (35). All templates were prepared in technical triplicate. Template amplification was set up and run on a QuantStudio™ 5 system (PCR System, Applied Biosystems™, Madrid, Spain) using the following cycling conditions on a fast-cycling mode with a comparative Ct type: a cycle of 95 °C for 20 s (enzyme activation) and 40 cycles each of 95 °C for 1 s (denaturation) and 60 °C for 20 s (annealing and extension). The Ct values acquired were inverted by subtracting from 40, which was the maximum number of cycles employed (36).

### 2.7 Statistical analysis

The normality of the data obtained was verified using a Kolmogorov-Smirnov test. Group means for the EVs characterization data (particle size and concentration) and inverted Ct of the miRNAs were compared using a multiple paired *t*-test at a 5% level of significance. Results were visualized using GraphPad Prism 10.2.3 for Windows (GraphPad Software).

### 2.8 Functional analysis

**Figure 1:**
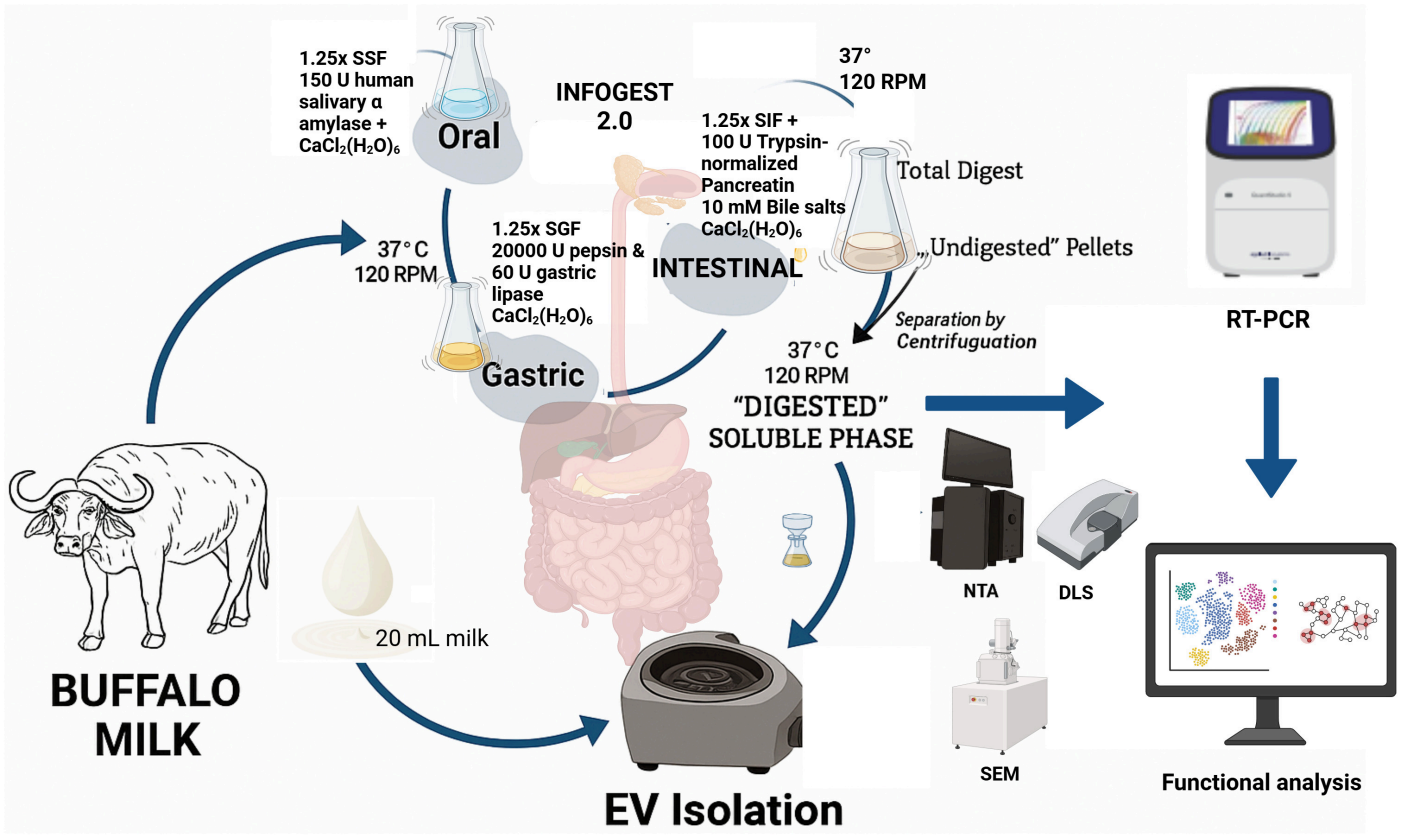
Protocol summary of gastrointestinal digestion, extracellular vesicle isolation, and miRNAs detection in buffalo milk.

Target prediction and enrichment analysis of the miRNAs were performed based on their expression levels in the BuM-EVs after simulated gastrointestinal digestion. Hence, miRNAs approaching undetectable levels were excluded. Validated target prediction was performed using multiMiR (37), clusterProfiler (38) and accompanying packages on RStudio (39) using default parameters. The target genes were used for the Gene Ontology (GO) and KEGG enrichment analysis. The Reactome enrichment was analyzed based on targets involved in the top 20 KEGG pathways, while the interaction between the proteins (PPI) was visualized using the full STRING network at a medium interaction score confidence, i.e., 0.4 (https://string-db.org/) (40) to understand the positioning of the hub proteins. Additional plots for visualization were generated using the SRPlot tool (41). Also, the PPI network was clustered using k-means and the top 20 nodes degree from each cluster (where possible) was used in plotting a circular plot on the Cytoscape software 3.10.3 (42). All the experimental procedures are summarized in Figure 1.

## 3 Results

### 3.1 Buffalo milk extracellular vesicle properties

**Figure 2:**
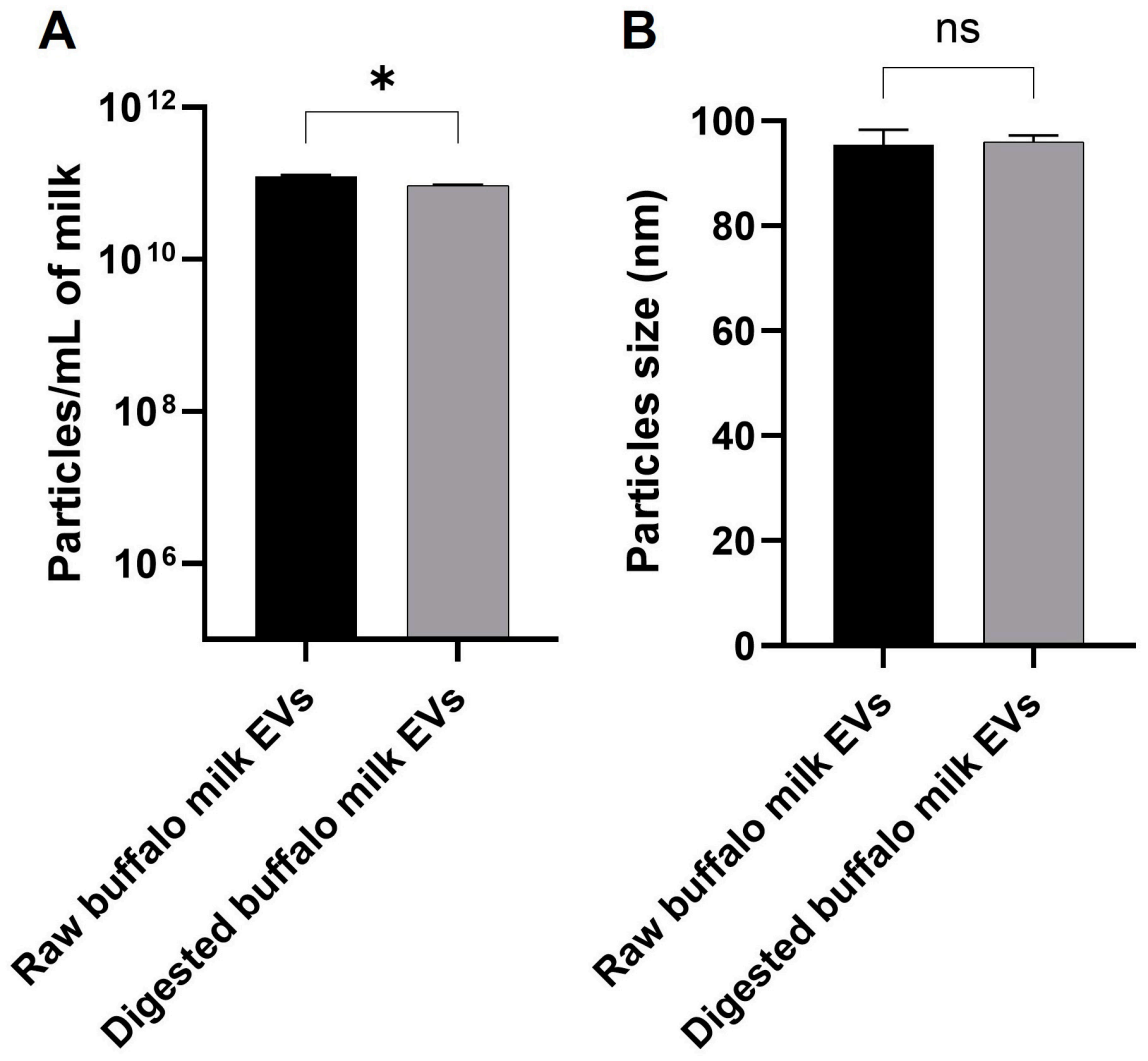
Nanoparticle tracking analysis of buffalo milk EVs by NS300. **(A)** Comparison of particle concentration in raw and digested buffalo milk EVs involved a minimum of four × 60-s videos recorded for each sample. **(B)** Comparison of particle size in raw and digested buffalo milk EVs. Three replicates of each sample were analyzed by NTA independently and presented as mean bars ± SEM. The significant *p*-value is reported; * indicates *p* < 0.05; n.s. indicates not significant.

**Figure 3:**
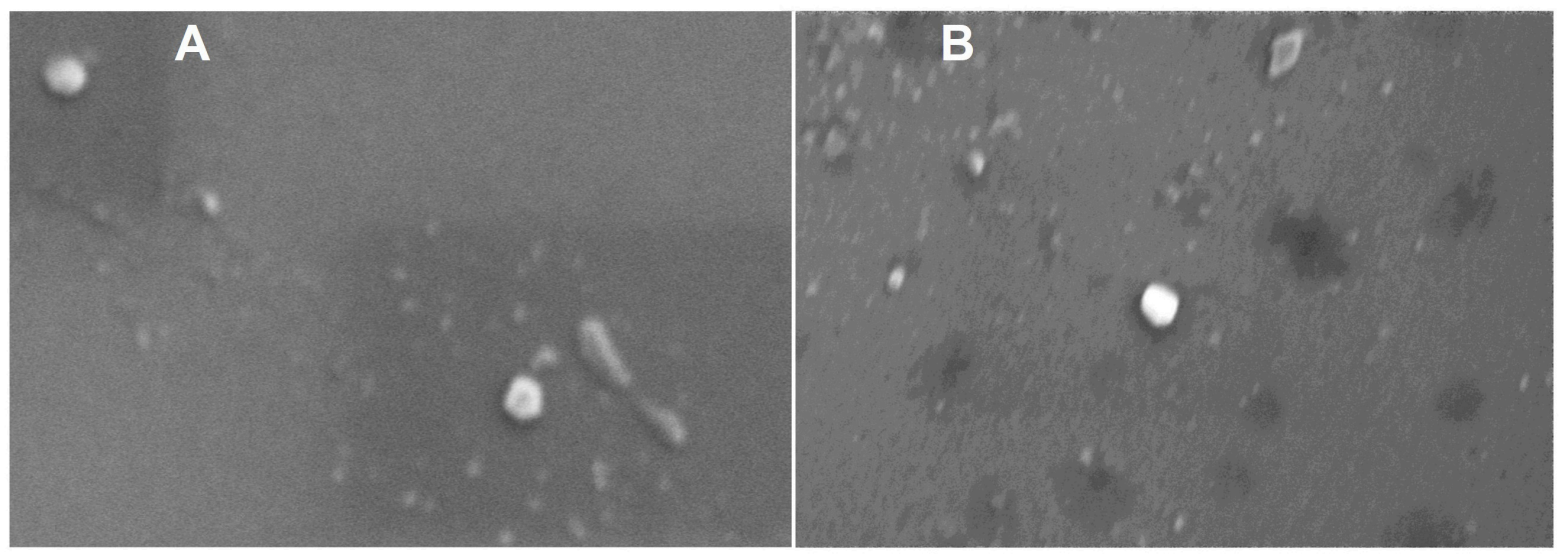
Scanning electron microscopy of raw **(A)** and digested **(B)** buffalo milk EV isolates.

The phase plot and frequency shift of the preliminary dynamic light scattering measurements (Supplementary Figure S1) for both the raw and digested BuM-EV isolates indicate an appropriate signal shape and resolution, which suggests good signal quality typical of well-dispersed colloidal systems with a monodisperse attribute. The system tended toward a relatively more stable colloid after digestion by the reduction of the zeta potential from −12.41 mV to −18 mV. The BuM-EVs exhibited a size range mainly between 30 and 200 nm. Specifically, EVs isolated from raw BuM exhibited a diameter size of 94.4 nm, while for digested milk EVs, the diameter was 96 nm (Figure 2A) (Supplementary Figure S2). Hence, no statistical differences were observed between EV samples in raw and post-digested milk. On the other hand, particle numerosity differed significantly (*p* < 0.05) after gastrointestinal digestion, as the EVs in raw milk (1.2 × 10^11^ ± 5.3 × 10^9^ particles/mL) were higher compared to those in digested milk (9.53 × 10^10^ ± 1.2 × 10^9^ particles/mL) (Figure 2B). The SEM also provided morphological details of the particles, showing vesicle-like lipid-enclosed structures (Figure 3).

### 3.2 Expression of widely conserved EV-miRNAs in buffalo milk as a factor of gastrointestinal digestion

**Figure 4:**
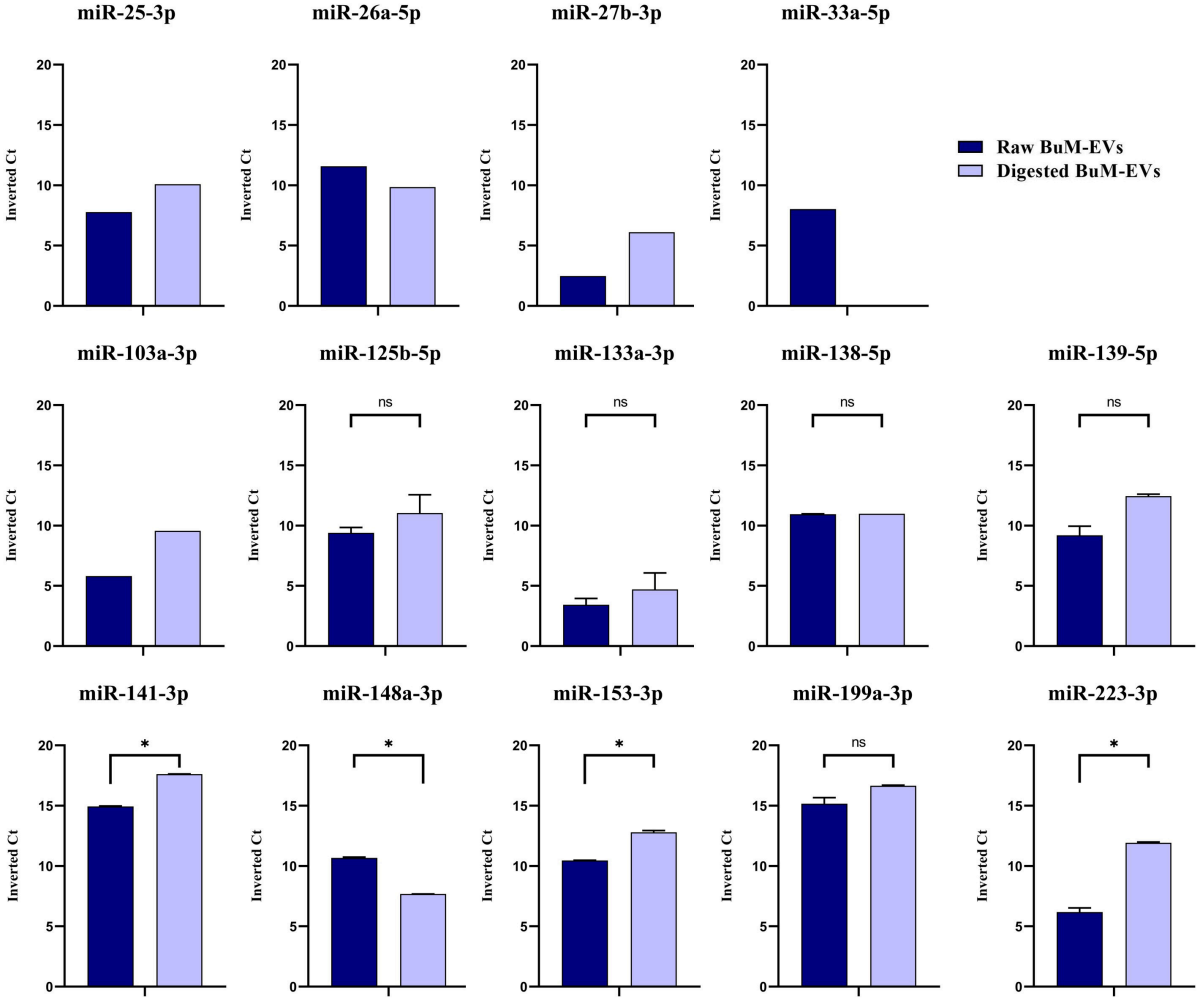
Expression levels of the widely conserved miRNAs in raw and digested buffalo milk EV isolates. Data are average ± SEM of 2 independent extractions. The data without error bars are excluded from comparison. * indicates *p* < 0.05; n.s. indicates not significant.

Based on the alignment statistics of *Bos taurus* miRNAs against their corresponding *homo sapiens* homologs, 17 highly conserved miRNAs, which exhibited optimal E-value as well as 100% identity, query coverage, and target coverage (mirbase.org). First, the presence of these miRNAs in raw BuM-EVs was ascertained. Three miRNAs, the miR-10a-5p, miR-24-3p, and miR-130a-3p, were undetectable. The average of the inverted Ct values indicated that miR-199a-3p (15.16) and miR-141-3p (14.94) were the most expressed among the detected miRNAs (36.4). Subsequently, the expression of these miRNAs in digested BuM-EVs was compared with the raw sample. The expression levels indicated that most miRNAs are largely stable. However, the expression levels of miR-148a-3p dropped notably, miR-33a-5p was undetectable post-digestion, while miR-141-3p, miR-153-3p, and miR-223-3p increased (Figure 4). Hence, only 13 of the miRNAs are expressed in the digest EVs, and as such, the remaining 4 (miR-10a-5p, miR-24-3p, miR-130a-3p, and miR-33a-5p) were excluded from the subsequent functional analysis. Similarly, the digestion influences the ranking of the miRNAs surviving the gastrointestinal digestion in terms of their abundance, although the two most abundant miRNAs were minimally affected (Table 1).

**Table 1:** Ranking of the miRNAs in terms of abundance pre- and post-digestion.

**Rank**	**Raw**	**Digest**
1	miR-199a-3p	miR-141-3p
2	miR-141-3p	miR-199a-3p
3	miR-26a-5p	miR-153-3p
4	miR-138-5p	miR-139-5p
5	miR-148a-3p	miR-223-3p
6	miR-153-3p	miR-125b-5p
7	miR-125b-5p	miR-138-5p
8	miR-139-5p	miR-25-3p
9	miR-33a-5p	miR-26a-5p
10	miR-25-3p	miR-103a-3p
11	miR-223-3p	miR-148a-3p
12	miR-103a-3p	miR-27b-3p
13	miR-133a-3p	miR-133a-3p
14	miR-27b-3p	miR-33a-5p

### 3.3 Enrichment analysis of the surviving widely conserved EV miRNA cargo

**Figure 5:**
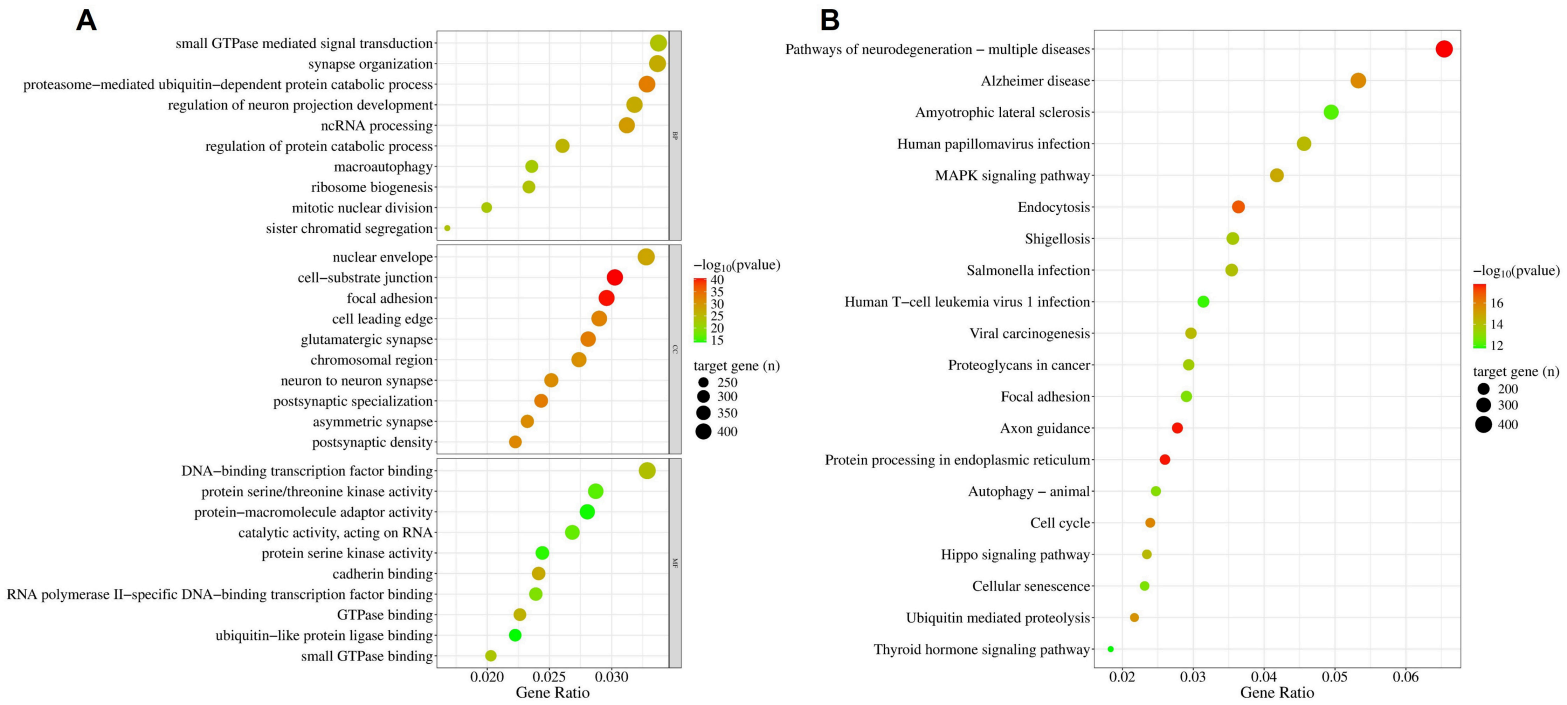
Enrichment of targets of simulated gastrointestinal stable BuM-EV miRNAs. The Gene Ontology **(A)** and KEGG **(B)**.

Using the multiMiR package, a total of 14,340 validated targets were identified from the 13 miRNAs that survived gastrointestinal digestion. The biological process of the gene ontology (GO) (Figure 5A, upper panel) indicates the enrichment of targets associated with protein turnover (macroautophagy and catabolism) and gene expression regulation (ncRNA processing and ribosome biogenesis), particularly within the context of neural development (synapse organization and neuron protection regulation) and intracellular signaling (small GTPase-mediated signal transduction). Their molecular function (Figure 5A, lower panel) also supported these processes through elements of ubiquitination, transcription regulation, RNA-related catalytic processes, cell adhesion (cadherin binding), and kinase/GTPase activity. The cellular location (Figure 5A, middle panel) of the targets gives a strong indication of their neuronal orientation and synaptic structures (glutamatergic synapse, neuron-to-neuron synapse, postsynaptic specialization, asymmetric synapse, postsynaptic density) and migratory or dynamic propensities (cell adhesion and cell-leading edge) while being transcriptionally active (chromosomal region).

Pathways essential to neuronal pathways are among the most enriched (Figure 5B). Particularly, pathways of neurodegeneration, Alzheimer's disease, and axonal pathfinding are among the top 20 pathways. Another enriched pathway which may be involved in neurodegeneration is ubiquitin-mediated proteolysis. However, the most represented pathways are those associated with viral and bacterial infections. Hence, the presence of “Human papillomavirus infection,” “Viral carcinogenesis,” “Yersinia infection,” “Salmonella infection,” “Shigellosis,” and “Human T-cell leukemia virus infection” suggests a high association of the targets with host-pathogen and immune response. This response may also involve pathways like endocytosis and MAPK signaling, which are equally enriched. Alongside other heavily targeted pathways, such as focal adhesion, axon guidance, and proteoglycans in cancer, the MAPK signaling pathway can be vital in regulating cell adhesion, morphogenesis, and extracellular matrix remodeling.

**Figure 6:**
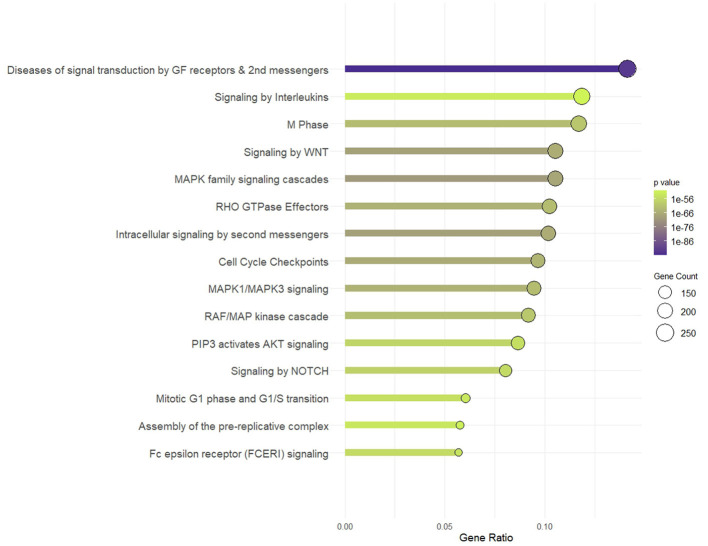
Reactome pathway. Pathway enrichment of targets of simulated gastrointestinal-stable BuM-EV miRNAs.

The target genes involved in the top 20 enriched KEGG pathways, which constituted 14.7% of all targets, were analyzed using Reactome pathways, yielding pathways critical to immune response, cell cycle regulation, intracellular communication, and cellular signaling networks (Figure 6), with an emphasis on neural and developmental processes, the dysregulation of which can result in diseases of signal transduction. These include interleukin signaling, which is particularly relevant in cytokine-related immune response signaling; Fc epsilon receptor (FCERI), an indicator of immune cell activation and potential involvement of IgE-mediated hypersensitivity mechanisms; and NOTCH signaling. The MAPK signaling axis, including MAPK family signaling cascades, MAPK1/MAPK3 signaling, and the RAF/MAP kinase cascade, which are widely recognized for their involvement in immune cell activation, differentiation, and cytokine production, was similarly enriched. Members of a regulatory pathway of cytoskeletal dynamics, RHO GTPase effectors, are equally enriched, aligning with the localization of the targets in synaptic and adhesion components.

### 3.4 Protein-protein interaction of predicted targets

**Figure 7:**
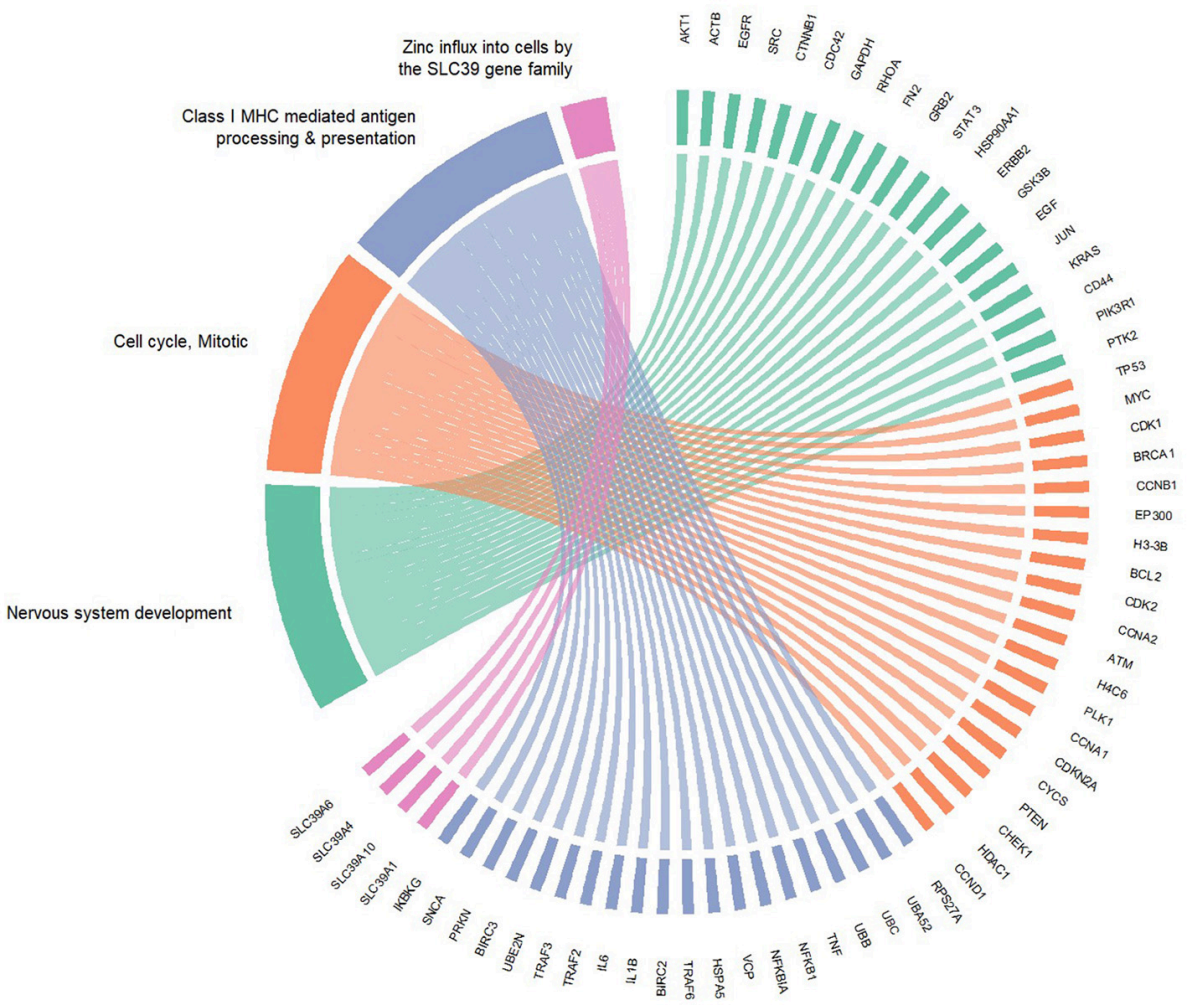
Cluster connections among the hub proteins of the identified clusters. Green — cluster 1. Orange — cluster 2. Blue — cluster 3. Pink — cluster 4.

Using the STRING database, a PPI was constructed from the enriched components of the Reactome pathways. The network identifies TP53, AKT1, ACTB, EGFR, and CTNNB1 (Table 1) as the hub proteins, with each interacting with at least 31.9% of the 1,799 network components. TP53 is the protein with the highest node degrees, having edges connected to about 41% of the interaction cloud. A 4-unit κ-means clustering of the network identified two important annotated clusters: Cluster 1, “*Nervous System Development*”, which holds more than half of the full PPI network, and another (Cluster 3) which is relevant to immune regulation, termed “*Class I MHC mediated antigen processing & presentation*,” accounting for 22.4% of all nodes in the network. The hub proteins of the latter were RPS27A, UBA52, UBC, UBB, TNF, and NFKB proteins. Cluster 2, which is the second largest, is annotated as mitotic cell cycle, with TP53 as its central node (Table 2). The smallest of the clusters accounts for only four members, which are recognized as an important machinery of zinc influx into cells (Supplementary Table S3). A circular plot (Figure 7) of the hub proteins of the clusters 1 in green, 2 in orange and 3 in blue indicates dense intra- and inter-cluster connections between the 3 clusters, indicating the pink cluster (responsible for zinc influx) a cluster with functional independence.

**Table 2:** Hub proteins of the full PPI network and the 3 main clusters.

**Node**	**Node degrees**	**Node**	**Node degrees**	**Node**	**Node degrees**	**Node**	**Node degrees**
***Full network***		***Nervous system development (Cluster 1)***		***Cell cycle, Mitotic (Cluster 2)***		***Class I MHC mediated antigen processing & presentation (Cluster 3)***	
TP53	740	AKT1	417	TP53	236	RPS27A	201
AKT1	714	ACTB	373	MYC	188	UBA52	195
ACTB	674	EGFR	367	CDK1	167	UBC	194
GAPDH	629	SRC	362	BRCA1	164	UBB	189
EGFR	601	CTNNB1	349	CCNB1	160	TNF	164
CTNNB1	574	CDC42	311	EP300	160	NFKB1	149
MYC	566	GAPDH	311	H3-3B	159	NFKBIA	140
SRC	533	RHOA	297	BCL2	152	VCP	138
UBC	529	FN1	269	CDK2	150	HSPA5	126
RPS27A	528	GRB2	262	CCNA2	145	TRAF6	123
HSP90AA1	525	STAT3	257	ATM	140	BIRC2	118
UBA52	516	HSP90AA1	253	H4C6	139	IL1B	117
UBB	508	ERBB2	252	PLK1	137	IL6	116
PTEN	491	GSK3B	250	CCNA1	128	TRAF2	116
TNF	486	EGF	248	CDKN2A	125	TRAF3	115
JUN	467	JUN	246	CYCS	124	UBE2N	113
BCL2	465	KRAS	245	PTEN	124	BIRC3	110
HSP90AB1	461	CD44	230	CHEK1	123	PRKN	110
STAT3	443	PIK3R1	230	HDAC1	122	SNCA	103
MAPK3	439	PTK2	226	CCND1	121	IKBKG	102

## 4 Discussion

Although a substantial level of attention is being given to the fate of EVs and their miRNA content in cow milk as a function of gastrointestinal digestion, the same level of scrutiny is not being given to other milks. Particularly, given the contribution of buffalo milk to global milk production, which has increased in recent years to become the second highest contributor (15% of global supply) (43), it is important to demonstrate what results from its EVs fraction post-digestion. This understanding is vital to both cross-species transfer and the functionality of dietary EVs and their molecular cargoes.

In concordance with previous studies (44–46), a combination of DLS, NTA, and SEM of the isolated EVs from raw buffalo milk jointly described lipid-bilayered, spherical-shaped nanostructures in the range 30–200 nm. Other biophysical parameters, like zeta potential and frequency shift, suggest a monodisperse and fairly stable colloid in the range of those reported by Joshi et al. (47) despite employing a different isolation technique. With this, we subjected the milk to simulated gastrointestinal digestion and examined the EVs extracted from the soluble fraction of the digest. Similar parameters of nanovesicular properties were obtained, although diminishing in numerosity, indicating that the EVs remained intact despite harsh digestive conditions. This attribute of the lactary product may be common to mammary milk, as evidenced by similar reports from human (48) and cow (49) milk when a mammal's gastrointestinal apparatus is simulated.

The miRNAs conservation across species is an interesting phenomenon which, coupled with the possible conservation of their target genes, may be a basis of “*dietary-nutrients miRNAs”* and their ability to produce biological effects following ingestion. Hence, the relevance of dietary miRNA is not only tied to their presence in food but also their survival in the digestive environment and potential to have targets in the intestinal mucosa of ingesting organism. However, it would be quite bold to assume that their expression is conserved due to the conservation of their sequence in the producing species (50). Similarly, an inquiry into the possible composition of these miRNAs as part of paracrine communication in EVs is of necessity, since such communication may be indicative of more complex processes, as in the case of mastitis in buffaloes (30). This study identified 14 widely conserved miRNAs: miR-25-3p, miR-26a-5p, miR-27b-5p, miR-33a-5p, miR-125b-5p, miR-130a-3p, miR-133a-3p, miR-138-5p, miR-139-5p, miR-141-3p, miR-148a-3p, miR-153-3p, miR-199a-3p and miR-223-3p, in BuM-EVs, which exhibited perfect alignment with their human homologs. A previous study, through small RNA sequencing, found the expression of all but one (bta-miR-138) (51) in raw BuM-EVs. The miR-148a-3p and miR-26a-5p, which are consistently expressed among the top ten most abundant miRNAs both in buffalo milk exosomes (51) and in whole milk (52), are equally highly expressed in this study. What's more, most of these miRNAs are largely stable after gastrointestinal digestion especially the miR-141-3p, miR-153-3p, and miR-223-3p displaying significant enrichment and apart miR-133a that is totally losted. This is probably due cause some EVs are lost during digestion the lipid bilayer of those EVs' envelope is destroyed. This necessitated the elucidation of their potential targets after possible absorption. For a number of miRNAs, a relatively higher expression was observed after digestion. We observed a significant reduction in the number of particles in the digested samples, indicating possible rupture of some EVs in the course of gastrointestinal digestion despite the retention of the size of the surviving EVs. This may afford the EV-related miRNAs in digests more accessibility to extraction. Although there is a minimal variation in the extraction recovery, minimal differences can exert significant differences in miRNA detection (53). Hence, the simulated digestive fluids could exert changes in the matrix (such as EV sensitivity to degradation), resulting in higher detection of EV-related miRNAs post-digestion.

The main speculation following the discovery of miRNAs in human breast milk was their potential role in modulating innate immunity and sustaining immunotolerance (6, 54), a function that could be conserved across other mammals (52, 55), as it is evident for human milk (56, 57) and might be possible for different types of milk. Exosomal miRNAs are important in guiding the intestinal immune development of dairy animal offspring and may offer this role through the stimulation of intestinal epithelium viability, proliferation, and stem-cell activity (58, 59); reduction of myeloid and lymphoid cells activation; and cytokine production (60, 61), while modulating the composition of microbial populations (62). The potential targets of BuM-EV miRNAs in this study are enriched in processes controlling protein turnover, which is essential in immune cells, where a rapid increase in growth and size demand massive energy and amino acids (63). However, the neurodevelopmental regulation enrichment suggests that their activities extend beyond the blood-brain barrier, as documented in another dietary source (64). Interestingly, the absence of extracellular regions among the most-enriched compartments suggests that the functional significance of the miRNAs may manifest mainly after cellular internalization. Endocytosis is a major route of EV cargo release (65), and as one of the main enriched pathways, also corroborates the intracellular functionality of EVs and their cargoes.

Infection-related pathways accounted for a majority of the most enriched functions of the gastrointestinal stable BuM-EV miRNAs. Studies have demonstrated the potential of EVs to regulate host-immune response (66–68), and MAPK is a signaling pathway usually employed in such (69), even by EVs (70). Other potential pathways identified in this study include the FCERI and NOTCH1 signaling, which are involved in IgE-mediated hypersensitivity mechanisms and T-cell lineage commitment and maturation, respectively (71–73). The targeting of GTPase-related proteins and ubiquitin ligases further supports the notion that milk EVs modulate immune cell trafficking, endocytosis, and antigen processing, particularly by dampening overactive immune responses (74). Nevertheless, the principal localization of the targets in cell-substrate junction, focal adhesion, and cell-leading edge, which are hubs related to axonal pathfinding (75, 76), emphasizes roles in structural remodeling and dynamic cell–cell communication, likely important during neurite extension and synapse formation (77). Coupled with the enrichment of targets in similar pathways and terms, these not only shed light on the roles of these milk miRNAs in contributing to neural development and synaptic plasticity (78, 79) but also on the modulation of the interaction between the immune and the nervous systems.

The PPI network revealed a major component of immune surveillance—the MHC Class I-mediated antigen presentation and processing. This machinery involves four main steps: peptide generation and trimming by the proteosome, peptide transport, MHC class I complex assembly, and antigen presentation on the cell surface to CD8+ cytotoxic T lymphocytes (80). The hub proteins of the cluster are ubiquitin pathway proteins, which are essential for generating peptides for MHC I, such as RPS27A, UBA52, and UBC; inflammatory signaling proteins that activate transcription of MHC I components, e.g., TNF, NFKB1, TRAF6; and protein folding and ER stress modulators, such as HSPA5 and VCP (81–83). Generally, the cluster presents a strong pro-inflammatory signature (such as IL1A, IL1B, IL18, TNF) and multiple Toll-like receptors (TLRs) alongside their downstream adaptors and transcription factors, confirming the involvement of innate immune pattern-recognition pathways where TLRs and inflammasome sensors modulate relevant pro-inflammatory cytokines production (84). Hence, the targeting of these components by the digestion-resistant EV miRNA cargo can offer anti-inflammatory effects. Also, the ubiquitin-proteasome processing and ER stress response signaling targets identified in the cluster can shape cytokine receptor turnover and buffer stress response during inflammation (85, 86), for instance, through the formation of unfolded protein response (UPR) in the ER and its cytosolic equivalent [aggresome-like induced structures (ALIS)], which can prevent excessive MHC 1 antigen presentation (87, 88). Similarly, antigen handling and cytokine control are used by autophagy in immune cells to prevent inappropriate activation (89). However, the more extreme form of programmed cell death, apoptosis, is documented to be employed by BuM-EVs through ER stress exacerbation, potentially by miR-27b (16), which is one of the 13 miRNAs surviving gastrointestinal digestion in this study. In immune contexts, this serves as a mechanism of immune homeostasis and tolerance (90). Since the ER stress and MHC I complex assembly impact each other (82, 91–93), there is potential for this cross-linkage to be modulated by the milk EV miRNAs, for instance, in reducing MHC I expression, and therefore, pro-inflammatory cytokines (94), given that the internalized exosomes at target cells are targeted to the ER, which is a site of nucleation of mRNA splicing and silencing machinery (19, 95). Similarly, there is also a possibility of cross-presentation from the lysosomal pathway (96), and some proteins involved in the cytosolic route (e.g., SEC61, VCP) are found in the identified cluster. Cytokine signaling and the overarching immune activities are increasingly recognized not only as an immune modulator but also as a regulator of neural development and synaptic function. Alterations in the expression of key signaling pathway members—such as IL6, TNF-α, and IL-1β–can influence the balance between excitatory and inhibitory transmission during development and injury responses (97, 98), as well as contribute to neuroinflammation or microglial activation (99), synaptic pruning, and homeostatic plasticity (100). The presence of an annotated cluster vital to nervous system development and the interconnection between the hub proteins of the clusters in this study further illustrate the relationship between the neural and immune systems and the role of dietary miRNAs in its modulation. Hence, milk may confer a neuroinflammatory regulatory advantage to the offspring being fed through the miRNA cargo of its EVs. These observations are comparable with the potential activity offered by human breastmilk EV miRNA fraction (101, 102), indicating such processes as core regulatory hubs consistently targeted by the mammal's milk EV miRNAs. Another identified cluster, comprising four proteins from the SLC39 gene family, is typically involved in acute-phase responses associated with inflammation and infection (103). Overall, these results highlight the promising potential of the widely conserved miRNA cargoes of BuM-EVs to regulate neural and immune processes following gastrointestinal digestion, highlighting potentially important targets for subsequent *in vitro* and *in vivo* inquisitions. Given the absorbability, bioavailability, systemic distribution, and biological actions at target sites (23, 104–106), this supports the broader case for dietary miRNAs as biologically active molecules, warranting further experimental investigation in this direction. The presence of widely conserved miRNAs in buffalo EV milk and in general in food of animals origin (46, 107–109) could design them as micronutrients (110) having immunomodulatory properties? For sure, nature does not do anything without a reason.

## 5 Conclusion

Based on the best-aligning of 17 widely conserved miRNAs with human homologs, we demonstrated the digestive stability of 13 miRNAs in buffalo milk EVs, thereby fulfilling a prerequisite for the biological efficacy of food-derived biomolecules. The validated targets of these miRNAs are associated with neuromodulatory and immune system functions, particularly through antigen presentation and processing. These findings suggest that buffalo milk, via its broadly conserved miRNA fraction, may exert biological activity following gastrointestinal digestion. However, confirming this possibility requires further *in vitro* and *in vivo* studies to investigate the absorption and functional impact of buffalo milk EV-miRNAs.

## Data Availability

The raw data supporting the conclusions of this article will be made available by the authors, without undue reservation.
