# Supplementary material for: Widely conserved miRNAs in buffalo milk extracellular vesicles survive gastrointestinal digestion and potentially target neural and immunomodulatory contexts
Source: Front Nutr. 2025 Oct 16;12:1685349. doi: 10.3389/fnut.2025.1685349 (PMC12574709; doi:10.3389/fnut.2025.1685349)
Supplement: Supplementary file 1 [file Table_1.docx]

***Supplementary Material***

**Table S1**. Alignment of the 17 microRNA conserved in human (hsa) and bovine (bta) from miRbase.

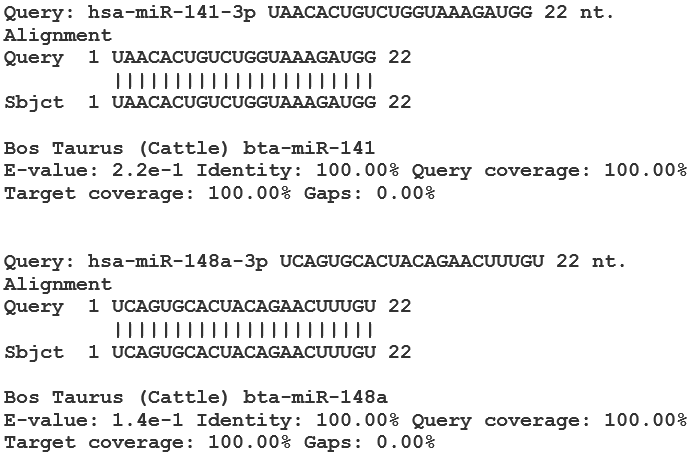

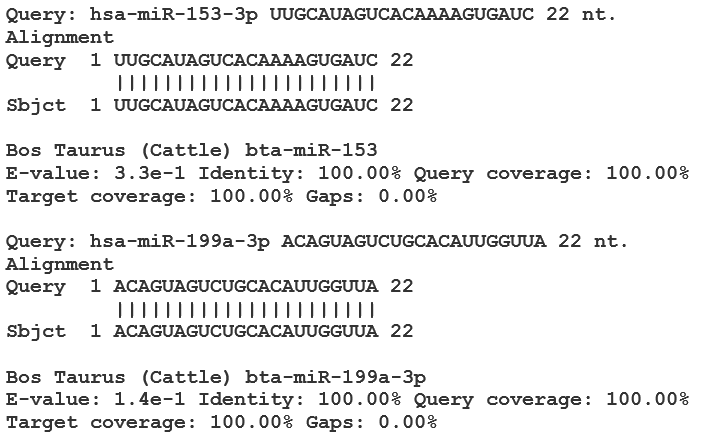


**Table S2**. Taqman™ Probes used in this study. Annotation and mature sequence.

| # | Probe ID | miRBase Annotation | Mature Sequence (5’-3’) |
| --- | --- | --- | --- |
| 1 | 479241_mir | miR-10a-5p | UACCCUGUAGAUCCGAAUUUGUG |
| 2 | 477992_mir | miR-24-3p | UGGCUCAGUUCAGCAGGAACAG |
| 3 | 477994_mir | miR-25-3p | CAUUGCACUUGUCUCGGUCUGA |
| 4 | 477995_mir | miR-26a-5p | UUCAAGUAAUCCAGGAUAGGCU |
| 5 | 478270_mir | miR-27b-5p | UUCACAGUGGCUAAGUUCUGC |
| 6 | 478347_mir | miR-33a-5p | GUGCAUUGUAGUUGCAUUGCA |
| 7 | 478253_mir | miR-103a-3p | AGCAGCAUUGUACAGGGCUAUGA |
| 8 | 477885_mir | miR-125b-5p | UCCCUGAGACCCUAACUUGUGA |
| 9 | 477851_mir | miR-130a-3p | CAGUGCAAUGUUAAAAGGGCAU |
| 10 | 478511_mir | miR-133a-3p | UUUGGUCCCCUUCAACCAGCUG |
| 11 | 477905_mir | miR-138-5p | AGCUGGUGUUGUGAAUCAGGCCG |
| 12 | 478312_mir | miR-139-5p | UCUACAGUGCACGUGUCUCCAGU |
| 13 | 478501_mir | miR-141-3p | UAACACUGUCUGGUAAAGAUGG |
| 14 | 477814_mir | miR-148a-3p | UCAGUGCACUACAGAACUUUGU |
| 15 | 477922_mir | miR-153-3p | UUGCAUAGUCACAAAAGUGAUC |
| 16 | 477961_mir | miR-199a-3p | ACAGUAGUCUGCACAUUGGUUA |
| 17  18 | 477983_mir  [478293_mir](https://www.thermofisher.com/order/genome-database/details/microrna/478293_mir) | miR-223-3p  cel-miR-39-3p | UGUCAGUUUGUCAAAUACCCCA  UCACCGGGUGUAAAUCAGCUUG |

**A**


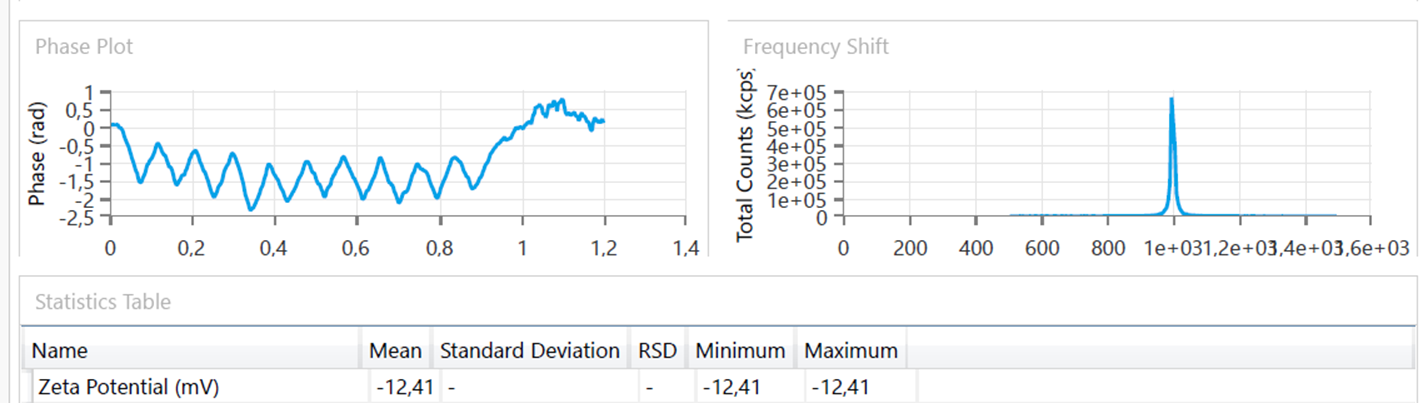


**B**


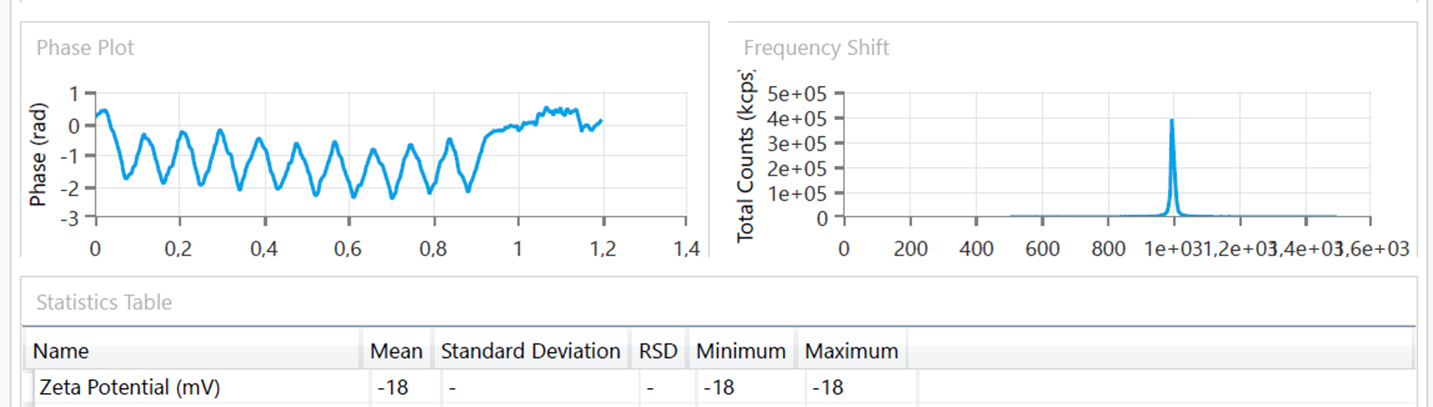


**Figure S1.** **Zeta Potential of Buffalo Milk EVs**. Representative graphs and statistics of EVs isolated by raw (**A**) and digested (**B**) buffalo milk analysed by DLS.

#


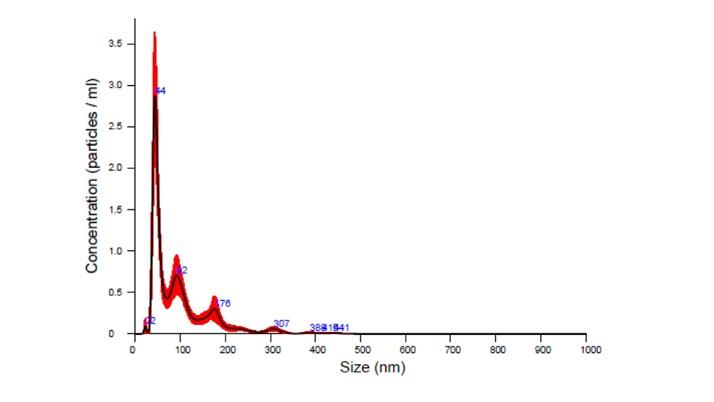

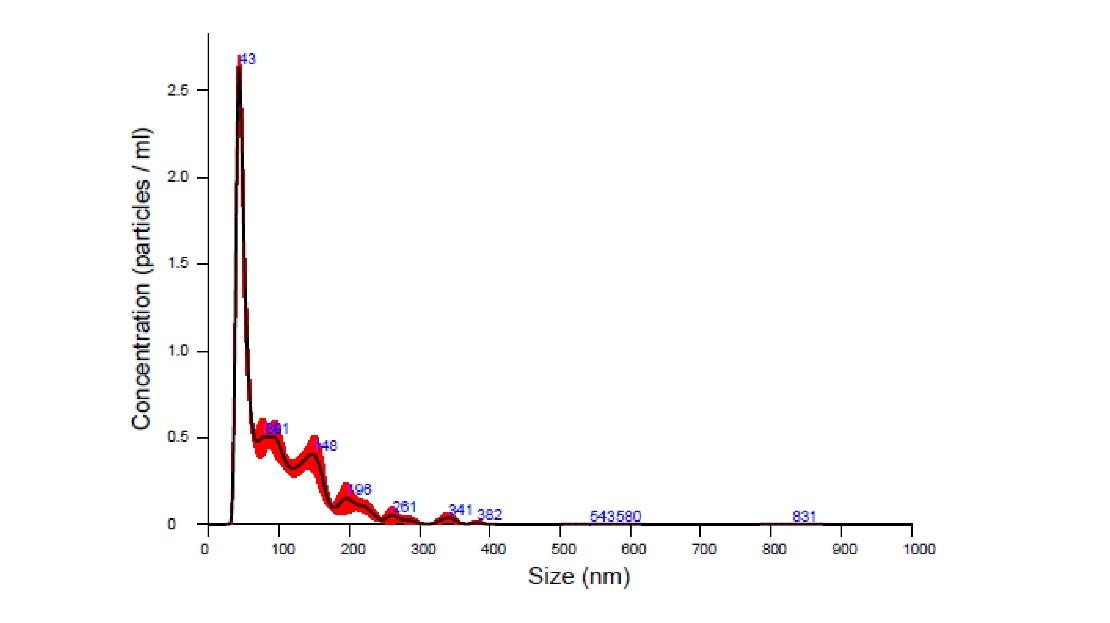


**Figure S2.** Representative graphs of the heterogeneous population of EVs in raw (**A**) and digested (**B**) buffalo milk analysed by NTA.

**Table S3**. The description of the clusters in the protein-protein interaction network.

| **Cluster #** | **Cluster color** | **Gene count** | **Primary description** | **Secondary description** | **Protein names** |
| --- | --- | --- | --- | --- | --- |
| 1 | Green | 913 | Nervous system development | - | NRAS, RAF1, BRAF, PRKCB, JAK1, PIK3CB, AKT3, ADCY7, CAMK2A, CALM2, CALM1, DLG4, CTNNB1, IGF1R, GNAI1, CACNA1A, ACTB, CDC42, ARHGAP35, DOCK1, PIK3C2G, PIP5K1C, PLCG2, PTPN11, SOCS1, KITLG, NOTCH1, TCF7L2, YAP1, FBXW7, FBXW8, DET1, APBB1, AKT1, MLST8, CLTC, AP2B1, RDX, AP2S1, GRIA3, NOS1, RYR3, PLCB3, FZD4, RYK, NTRK1, FRS2, FGF1, LPL, LDLR, CBLB, LCK, BAG1, HSPH1, HSPA8, TUBA1A, ACTG1, WASF2, CYFIP1, CYFIP2, NUP88, NUP62, CUL7, WTIP, DYNC2H1, DYNLL2, DYNC2LI1, DYNC1I2, DCTN2, DCTN4, KLC1, DPYSL2, GRIN2B, EFNB2, EPHB6, EPHA5, ROBO2, SLIT2, SRGAP2, ROBO1, PAK6, PAK4, ITGB3, COL9A3, ITGA7, LYN, GRIA1, AGAP2, PIK3R1, TGFBR1, RAN, NUP37, NUP153, NXF1, SNW1, PPIL2, ALYREF, RANBP1, TFDP2, MAML2, PSENEN, MAML3, WWC1, LATS1, TEAD2, TEAD1, WWTR1, STK3, TUBA1C, MYL12A, CD63, MME, SEC24B, HLA-DPB1, PTPRC, SEMA4D, PLXNB3, SEMA3E, UNC5C, DCC, NCK1, BRK1, CFL1, CLTCL1, ASAP1, SH3KBP1, SOS1, HRAS, PIK3CG, ARRB1, FLNB, LAMA2, CD44, TWIST2, FGFR2, VEGFC, ANGPT1, TYK2, PIAS1, PIAS2, ATP2A2, BDNF, CDK5, CAPN1, CAPNS1, CAST, LAMP2, EPN1, TGFA, PDGFC, FGFR1, FGF16, GDNF, PRKCA, ADCY5, ESR1, RXRA, MED24, NCOA2, CACNB4, RASGRP4, MAPK9, RHOA, RASGRF1, CACNA2D3, CACNA2D2, RAE1, SEH1L, LAMTOR3, CAPZB, PRNP, SEPTIN7, CSNK1D, NEDD4L, XPO1, FANCL, IGF2, SHC1, AP2M1, FNBP1, ARHGEF12, ROCK1, GAPDH, IRS2, GRB2, BCAR1, ARPC1B, ARPC4, DAPK1, E2F4, CUL4B, THRB, NCOA1, NFATC1, CXCR4, HLA-DPA1, PTPN6, ITGB2, ITGA6, TNXB, ITGA4, PARVB, ILK, ITGB1, COL4A4, SDC1, TUBB2A, DYNLL1, PPP3CC, NFATC2, MAPK8, MAP2K7, NLK, AKT2, CSNK1E, TPTEP2-CSNK1E, BTRC, GLI2, SLC39A7, RXRB, MED13, MYH6, ACTN1, CTNNA2, YWHAQ, AKT1S1, EIF4B, CAMK2B, CAMK2D, PPP3CA, STMN1, EPS15L1, AREG, SEC24A, SAR1B, RAB9A, DNM2, SNX9, WIPF1, WASF1, GIT1, EPHB2, EFNA4, EPHB1, PLXNA1, LAMA1, LAMB3, ITGA8, COL2A1, HLA-DRA, SEC31A, FOLR1, GPC3, UNC5D, SRC, SYK, PIK3CD, PRKCZ, CXCL12, BMP7, BMPR1B, RGMA, BMP2, IGF1, PDGFD, TEK, ANGPT4, HSPG2, FGF9, NTF3, SEC13, POM121, NUP58, NUP160, HSPA1L, DNAJA1, HDAC6, CTTN, ACTR2, DIAPH1, NRG4, NRG3, FYN, CD3E, HLA-DQB1, IRF9, MX1, RASGRP3, CACNA1I, CACNB1, CACNG7, DLG3, DLG2, PARD3, YWHAB, TBC1D4, RAB31, DUSP8, JUN, CUL4A, FBXO6, ARFGEF2, KLC2, KIF5A, KIF5C, DVL1, NOTCH4, HES5, PLAU, IGFBP3, HSPA1A, HSPA1B, HNRNPK, ABI1, NCKAP1, ABL1, EPHA6, EPHA7, EFNA3, EFNA1, THBS2, MMP9, CYBB, ITPR2, PLCB4, PPP3CB, PRKACB, ADCY3, GNAS, UBE2D1, IFNB1, IL6ST, CCN2, SDC4, COL4A1, DKK1, CKAP4, RTN3, RBL1, MTOR, RRAGC, RRAGA, L1CAM, SH3GL2, SRGAP3, AMPH, HLA-DRB5, STAT1, PIAS3, HSP90AB1, NOX1, VAV2, RAPGEF1, RAP1A, PLCE1, PIP5K1A, GSN, MYO6, RHOBTB2, KLHL13, GLMN, COP1, SEMA5A, PLXNA3, SEMA4A, PLXNA2, PAK3, GNG12, PAK5, RHOH, ATP2A3, RYR2, CACNA1E, CACNA1B, GNAI3, NGF, ARTN, SHC3, EPHA2, VAV3, ITGAL, BMPR2, ZFYVE16, BMPR1A, ACTR1A, SPTBN2, UBR4, SRSF3, NCBP1, NUP188, NDC1, TPR, RRBP1, COL6A1, THBS3, DUSP10, ARAP1, CAPZA2, FLNA, MED12, NCOA3, DCTN1, DNAJC6, EPS15, LDLRAP1, AP2A1, MSN, DLG5, PARD6B, SMURF1, UBE2D3, AMOT, LATS2, MOB1A, MOB1B, STK4, BAG2, DNAJC5, UBE2I, NUP214, NUP50, KPNA1, IFNAR2, PDGFA, FN1, COL18A1, COL4A2, LAMC3, PTK2, NEO1, ENAH, RAC1, ARHGEF2, DYNLT1, DYNLRB1, DYNC1H1, TUBB4B, MAPT, PSEN2, APH1A, EPHB4, HEY2, HEYL, RBPJ, CACNA1G, RYR1, ATP2A1, PLN, PRKACA, EZR, NF2, NF1, TGFBR2, TGFB2, MYLK, PKN1, MAP4K4, WNT7B, FZD9, WNT2B, FZD8, FZD5, FRAT2, NHLRC1, GRIN2A, GRIN1, CHRNA7, CHRFAM7A, APP, PSEN1, ERBB4, SCRIB, FZD6, WNT8B, CACNA2D1, MEF2C, TEAD4, TEAD3, TCF7, SMAD4, RET, HSP90AA1, ANXA2, PIK3C2B, EPHA3, SLIT3, SRGAP1, FES, PLXNA4, PLXNB1, SEMA4F, SLIT1, PDGFB, FGFR3, CRKL, IRS4, PRKCD, PLCB1, CAV1, HNRNPA2B1, SIAH1, GSK3B, SMAD3, HEY1, MFNG, RFNG, EGF, ERBB3, ERBB2, EGFR, NRG1, ADAM17, BACE1, RTN4, LAMP1, SND1, SEC24C, HLA-DRB1, HLA-DQA1, IGF2R, PLAUR, FBXO2, PDPK1, RRAGD, PRKAA2, SLC39A2, SLC39A3, STAT3, STAT5B, STAT5A, SOCS3, PKM, HNRNPA1, NUP43, SRSF7, NOS2, PAK2, LIMK1, LIMK2, GRM5, MTMR4, MTMR14, EPN2, AP2A2, PTCH1, SOX2, NUP98, DYNC1I1, TUBB, TUBA1B, TUBA8, TUBB6, KLHL9, RHOBTB1, ROCK2, ARHGEF1, RHOG, ARHGEF26, RHOB, TIAM1, MRAS, ARAF, CACNB2, CACNA1H, RASGRP1, TLN1, PARVA, GIT2, WIPF2, WASF3, ELMO1, RAC3, PARD6G, CRB3, CLTB, DAB2, DVL3, FZD2, DUSP5, STK11, PRKAA1, CRK, MET, EGR3, FOSL1, MAP3K11, PAK1, RHOJ, ARHGAP5, RHOD, RND1, UNC5B, NTN4, UNC5A, GNAI2, ADCY2, ADCY4, ADCY6, ADCY1, ADCY9, EIF4EBP1, CDK5R1, CAPN2, TERT, SMO, SHH, UBE3C, SMAD1, ZFYVE9, GCC2, NUP93, GLE1, NUP35, MED14, MED16, RXRG, FOS, NFATC3, ANGPT2, MED1, HDAC3, YWHAG, DDIT4, CAMKK2, RPTOR, EFNA5, EPHB3, AXIN2, WNT10B, LRP5, DDB1, STAT2, CTNNA1, FBXO22, CUL3, SKP2, PDCD4, TIMP3, COL1A2, ITGA11, TNR, ITGA3, LAMA3, LAMB2, COL6A2, PPID, DNAJA2, SEC23A, SEC24D, LMAN2, CD3D, CCR5, GRK2, GRK3, GNAQ, FZD1, WNT1, WNT9B, FZD7, ITGA5, ITGB5, ITGA2, COL6A3, ITGB6, ITGA1, IL15, VEGFB, INSR, IRS1, KIF5B, DCTN5, ACTR1B, ARPC2, ARPC5, NCKAP1L, ELMO2, PXN, RASA1, EPHA4, EPHA1, NTRK2, NRTN, PIK3CA, KIT, IFNAR1, FBXW11, RAPGEF2, SNX18, PIK3C2A, NCSTN, HDAC11, NCOR1, MED13L, MED27, MED30, ERCC8, FGF18, FLT1, FGF19, FGF7, WNT3A, WNT7A, WNT9A, TCF7L1, LEF1, WNT2, WNT5A, ICAM1, LUM, FGF2, NRP1, SEMA6D, KDR, SHC2, CBL, TUBB4A, DYNC1LI1, KRAS, CALM3, CAMK2G, CACNG2, CACNB3, CACNA1D, GRIA2, GRIN2C, GRM1, DPYSL5, ABLIM1, ABLIM3, RAC2, IQGAP1, ADCY8, GRIA4, CACNA1C, CACNG8, RASGRF2, GRIN2D, CACNG4, LIMD1, SAV1, AJUBA, ANK3, PLCG1, PDGFRB, PIK3R3, GAB1, ATF2, HDAC4, RANBP2, NUP205, HSPA4L, TSC1, MYL9, ITGA2B, ITGB7, TNC, SEMA3A, PLXNC1, SEMA7A, ITGA9, LAMC2, ITGAV, VWF, SAE1, PIAS4, BOC, EGR1, JUND, CLTA, EHD2, RAB10, KLC4, SMURF2, AXIN1, RBL2, PDGFRA, PLCB2, PRKCG, SLC9A1, CDH1, SMAD2, SMAD7, THBS1, SDC2, GPC1, NOTCH3, APH1B, NOTCH2, PRKCI, MPP5, NGEF, ACTR10, DCTN3, CAPZA1, ACTR3, DYNC1LI2, LMAN1, PREB, RRAS2, RASA2, GNA12, PPP5C, DNAJB1, ACTN4, PIP5K1B, PIK3R2, FLT3, U2AF1, U2AF1L4, NUP54, IRF1, OASL, DDB2, MED4, THRA, MED17, FDPS, NUP85, NUP133, NXT1, NUP210, NUP155, NUP107, DNAL4, SKP1, APC, CSNK1A1, LRP6, WNT10A, BMP4, JAG1, FLT4, EREG, YWHAH, PPP3R1, PFN1, VASP, SEMA6A, RRAS, RPS6KB1, RHEB, RRAGB, RBX1, UBA2, ARPC1A, WIPF3, NCK2, EFNB3, MMP2, TGFB1, ITGB8, COL1A1, LAMC1, LAMA5, LAMA4, MMP7, C3, APOE, IDE, CTSD, SERPINE1, HBEGF, GSK3A, TSC2, HDAC5, SNAI2, HGF, HPN, RAP1B, ARAP3, VCL, WASL, ARPC3, FNBP1L, PFN2, MAPK13, DUSP16, DUSP4, DUSP2, CD4, IL2RB, SOS2, LAMB1, ITGB4, CAV2, IBSP, DCN, NGFR, EFNA2, EFNB1, WNT3, FZD3, WNT16, FZD10, DKK4, DVL2, PARD6A, CACNG3, STUB1, TUBA4A, DCTN6, MGRN1, M6PR, PRPF19, NTN1, MYL7, MYL2, HES1, LFNG, HES7, AMH, CHRM3, ARAP2 |
| 2 | Orange | 479 | Cell Cycle, Mitotic | - | UBE3A, PLCD3, CCNA2, CCNH, H4C8, H3C6, H2BC6, H3C2, H4C5, H3C8, H3C7, H2BC15, H2BC3, H3C11, H4C9, H4C13, H3C1, H4C4, H2BC9, H4C1, H2BC14, MYC, H2BC17, H2BC11, NDUFV1, ATP5MC2, COX7B, NDUFA6, UQCR11, COX6B1, PFKM, HK1, RPS6KA1, NEFL, TRPM7, TRPC4, PLCD1, FAS, APAF1, IRF3, MCM3, ELOC, UBE2F, ANAPC16, HIF1A, NR4A1, DROSHA, CDC45, ORC4, CDKN2A, BRCA1, HDAC2, CHD4, H4C14, H4C15, GTF2A1, GTF2H3, DDX11, GTF2A2, H3C15, ANAPC4, CDC27, ANAPC15, CCNE2, LIN52, PPP2CA, DAPK3, AMBRA1, ATG13, COX4I1, CREB3L1, RPS6KA5, RPS6KA2, RAB5C, VPS11, VPS45, UBE2L3, VDAC3, ATP1A1, ATP1A3, TRPC1, EHD1, HDAC9, H2BC18, H2BC8, H4C3, H3C4, CHEK2, CDC7, WEE1, PPP2R2D, PPP2R5D, PPP2R5C, BCL2, BBC3, GPX8, CCS, ATP6V1C1, ATP6V0B, ATP6V0E1, ATP6V1F, UQCRB, NDUFC1, COX6C, COX7C, NDUFB2, NDUFA5, NDUFS1, TOMM40, CUL2, RAB1A, CHEK1, PCNA, CDKN1A, E2F5, CDKN1C, UBE2D2, MID1, HERC1, TP73, SIN3A, CUL5, TRIP12, CREB1, RELA, CDK1, MAD1L1, ANAPC13, HIPK2, FOXO1, BAD, YWHAZ, ATP5F1A, ATP5PF, NDUFA11, ATP5MC1, ATP6V0E2, MKNK1, RPS6KA3, CDC23, FZR1, PTTG1, STX10, SLC2A1, PFKP, SDHB, SDHD, UQCRQ, VDAC1, SLC25A6, H2BC13, STAG1, HDAC8, VPS16, RAB5B, RABEP1, ATG16L1, ATG9A, SUPT20H, BCL2L1, GPX3, HDAC1, TFDP1, CSNK2B, ID1, ETS1, TRPC3, PLCD4, MTMR3, ITPR3, TRPV4, PTEN, FOXO4, TXN, CDC20, ORC1, RBBP4, ATRX, RAD50, RAD1, RAD9B, SMC1B, SMC3, NDUFS5, VDAC2, BAK1, SLC25A5, ATP5PB, SDHC, MAX, ZBTB17, E2F2, CCND3, CDKN2D, E2F1, TRRAP, ATP6V1G1, ATP6AP1, ATP1B1, DNM1, PLA2G4A, PTGS2, FASLG, H2BC21, H3C10, H2BC10, H3-3A, MAP3K5, DUSP9, ETS2, RAB4A, WDR45B, BECN1, CDC14B, CDC14A, BUB3, ANAPC11, WWP2, GTF2B, GTF2E2, H4C11, H2BC7, H2BC12, HTT, ANK2, ATP1A2, UBE2C, CDC16, ANAPC1, TP53BP2, DLG1, PPP2R5E, FOXM1, PIK3R4, UVRAG, RUFY1, COL4A5, FOXO3, USP7, ATG7, DNM3, ATG101, CRTC2, ATP6V0A2, ATP6V1H, ATP5F1C, COX7A2L, NDUFA4, UQCR10, NDUFA12, NDUFV2, ATP6V0C, HMGB1, SP100, GPX7, GADD45A, SFN, PMAIP1, CREB3, PTPN5, ITPR1, CYCS, CYC1, COX5A, COX8A, NDUFS8, UQCRH, UQCRFS1, SMC1A, MRE11, ATR, KAT5, H2BC4, CBX3, KNL1, CDC25C, BIN1, HIP1, KPNA4, ACBD3, GORASP1, AURKB, MAP2K1, PPP2R1A, PPP2R2A, PPP2R1B, PFKFB2, HKDC1, BAX, BID, ULK1, VPS39, SH3GL3, PRKCE, PRKDC, RAD9A, CDC25A, TP53, ATM, ESCO2, PDS5B, PDS5A, BUB1, ANAPC2, HERC4, RAB5A, EEA1, MAD2L1, BIRC5, ELK1, SRF, CXCL8, ANG, SOD1, LIN54, LIN9, PLK1, MCM7, CDT1, YWHAE, RAD21, WAPL, CCNB2, BUB1B, RNF7, CREB3L2, WIPI2, MAML1, CRTC1, CRTC3, EP300, KAT2B, TADA3, H2BC5, CDKN2B, RB1, PML, GTF2E1, GTF2H2, GTF2H1, NIPBL, CDCA5, ESCO1, MCM6, MCM2, ORC5, CDK2, MAPK3, DUSP6, RAB7A, ATP6V1A, ATP6V0D1, ATP6V0A4, ATP6V1E2, ATP5PO, ATP5PD, NDUFS4, ATP5MC3, SLC25A4, UQCRC2, NDUFB9, NDUFS6, NDUFA9, SDHA, PFKL, HK2, PPP2R5A, SGO1, DBF4, ELOB, ANK1, TRPC6, TRPC5, CREB3L4, MECOM, UBE2S, MDM2, DAXX, HIPK1, NBN, HERC2, CCNA1, CDK6, ANAPC5, CCNE1, E2F3, CREBBP, TCF3, H3-3B, CCNB1, ESPL1, NDC80, MCM4, PKMYT1, RPS6KA6, EHD3, CSNK2A2, RCHY1, CDKN1B, CCND2, CDK4, CDKN2C, ATP1B3, FXYD2, ATP1B2, MAPK1, MAP2K2, ADAM10, PIK3C3, WIPI1, ATG10, VAMP8, VPS41, VPS33A, RAB33B, ALS2, ATP5F1B, ATP6V1B2, TCIRG1, ATP6V0A1, VAC14, ATG14, STX17, TRAP1, KPNA3, VHL, H4C6, GTF2H4, CDK7, TBP, NFYB, TUBG1, TUBG2, TWIST1, CCND1, CDC25B, ATP6V1E1, COX5B, ATP5F1E, COX6A1, TXN2, FBXO5, SIRT1, KAT2A, HDAC7, HDAC10, DUSP3, ID2, CSNK2A1, PPP2CB, PPP2R5B, POLB, HUS1, MAD2L2, RB1CC1, CDC6, ORC2, ORC3, ORC6, MCM5, MYBL2, UBE3B, SNAP29, YKT6, STX1A, VPS18, RAB9B, RBSN, ARL8A, ATP5F1D, ATP6V1D, UQCRC1, DDX5, ABLIM2, GORASP2, FIG4, STAG2, MAU2, CAT |
| 3 | Blue | 403 | Modification-dependent protein catabolism | Class I MHC mediated antigen processing & presentation | BCL10, MYD88, SNCA, MAP1LC3B, ATXN3, UBE2W, UBB, ADRM1, PSMC6, CYTH2, ITCH, IKBKG, MALT1, BIRC2, MAP3K14, PSMB3, RAD23A, PSMC3, NDUFAB1, UBC, HYOU1, DNAJC3, PRKCSH, ARF1, STING1, IRAK4, TRAF6, IKBKE, DDX3X, SEM1, ZFP36, UBA52, UBE2V2, UBE2E3, PSMD9, IKBKB, CASP1, GSDMD, CHMP2A, CHMP4A, RNF103-CHMP3, IST1, MYL12B, ATG12, MFN1, DDIT3, PDCD6IP, B2M, EXOC5, MAP2K4, WWP1, NEDD4, GABARAPL1, NSFL1C, PSMD6, VPS29, MAPKAPK5, MAP2K6, MAPKAPK3, BCAP31, VAPB, MFN2, NDUFA3, ARFGAP2, MOGS, TUSC3, DDOST, DNAJB11, HERPUD1, ERN1, TNF, LTA, PSMA1, PSMD13, PSMC2, CRYAB, SEPTIN2, CYTH1, PPP1R12A, IL6, IL1R1, TLR6, CD14, TRIM32, UBE2E2, HERC3, UBE4A, UBE2J1, BIRC6, HLA-B, HPSE, USP8, CHMP1A, SNF8, CHMP7, MAP3K1, PSMD14, TFRC, SLC11A2, SLC39A8, SLC39A14, PPP1CB, VPS28, VPS36, STAMBP, STAM, HLA-A, SQSTM1, KEAP1, NFE2L2, NDUFB6, RPS6, SEC61G, UBE2J2, DERL3, PDIA6, ERO1A, HLA-C, HLA-E, HLA-G, IRF7, MAP3K7, TAB3, TRAF3, TAX1BP1, OPTN, SHARPIN, TRAF1, TNFRSF1B, TLR4, TIRAP, NLRC4, CASP4, SELENOS, STT3A, DUSP7, GBF1, PINK1, UBQLN1, TAOK3, UGGT2, EDEM2, SYVN1, UBE2G1, RNF5, VTA1, MVB12B, PARK7, PRKN, PSMD4, PSMA7, PSMB2, NDUFS2, NDUFA8, NDUFA1, TAB2, IRAK1, CHUK, XIAP, UBE2A, ATF6, MBTPS2, ATF6B, NFKB2, PSMB5, VCP, SNX5, SNX2, TXNDC5, RAB11FIP2, CASP8, CASP7, ATG5, ATG4B, CGAS, TLR9, MAP3K3, NAE1, UBA3, UBE2Z, MAPKAPK2, PSMD12, RAD23B, PPP1R12B, MAP2K3, UBE2H, PPP1CC, PPP1CA, NLRP3, CTSB, ATG4A, WDR45, RAB39B, UBQLN2, CTSL, MBTPS1, MAN1B1, MAN1C1, MAN1A1, MAN1A2, GANAB, MAGT1, HSPA5, P4HB, ERO1B, RNF41, CASP9, NBR1, IL1R2, NDUFV3, CALR, CSF1, CASP3, RNF185, SEL1L, UBE2G2, UBA1, UBE2V1, TIFA, UBE2N, UBA7, CUL1, TBK1, TRADD, RBCK1, NPLOC4, SVIP, UBXN1, HUWE1, ATF4, RPS6KA4, MAPK11, HGS, CHMP6, VPS37A, VPS37C, CHMP4C, RAB8A, ARF4, ARFGAP1, MAPK7, CFLAR, RNF31, TNIP1, FADD, GABARAP, RAB11B, EXOC7, RPS6KB2, PSMC5, NFKBIB, PSMD2, UBA6, UBE2O, OS9, EDEM3, PSMD1, PSMB4, REL, PSMA5, RPS27A, RPS3, LRRK2, VPS35, CSF2, ZFP36L1, ZFP36L2, UBE2E1, UBE2Q1, UBE2Q2, UBE2M, HSP90B1, PDIA3, RPN1, SEC61A2, AMFR, TLR3, CSF1R, EIF2AK3, IL1RAP, IL18, MARCHF6, ATG3, ATG4D, ATG4C, CYTH3, ARF6, PLD1, PPM1B, PPM1A, SH3GL1, STAM2, VPS37B, CHMP3, NDUFA7, NDUFB8, NDUFC2, NDUFB11, ECSIT, NDUFB10, SLC1A2, UFD1, YOD1, DERL1, NGLY1, PSMC1, PSMD3, PSMB6, IL1B, BIRC3, TNFRSF13C, PSMB1, UBE2B, UCHL1, SNCAIP, UBE2L6, PSMD11, MAPK14, DUSP1, RAB11A, RAB11FIP3, UBE2R2, UBE2K, MAP3K8, TLR2, IL1A, PRKCQ, TRAF2, RIPK1, TNFRSF10B, VPS26A, ARF3, ARFGAP3, VPS4A, CHMP2B, SIGMAR1, CANX, EDEM1, UGGT1, NDUFA10, NDUFS3, SEC61A1, SSR1, DAD1, RPN2, SEC61B, EXOC2, EXOC4, HSPB1, NFKB1, EIF2S1, PSMA6, PSMB7, PSMD8, PSMA2, TAOK1, TAB1, RIPK2, NOD1, PYCARD, NFKBIA, RELB, LTBR, TANK, TICAM1, PLD2, NDUFB5, NDUFA2, NDUFB7, PSMD7, CHMP5, VPS4B, TSG101, VPS25, TNFSF10, EIF2AK2, XBP1, WFS1, RALA, HLA-DOA, HLA-F, TNFRSF10A, TNFRSF1A, PSMA3, UBE2D4, UBOX5, GABARAPL2, CHMP4B, PSMC4, ARF5, CYTH4, CDC34, PSMA4, RAB35, SNX3, PPP1R15A, NDUFB4, HSD17B10, IFT57, DERL2, ERLEC1, TRIM37, IQSEC3 |
| 4 | Pink | 4 | Zinc influx into cells by the SLC39 gene family | - | SLC39A1, SLC39A10, SLC39A4, SLC39A6 |
