# Supplementary material for: Widely conserved miRNAs in buffalo milk extracellular vesicles survive gastrointestinal digestion and potentially target neural and immunomodulatory contexts
Source: Front Nutr. 2025 Oct 16;12:1685349. doi: 10.3389/fnut.2025.1685349 (PMC12574709; doi:10.3389/fnut.2025.1685349)
Supplement: Supplementary file 2 [file Table_2.docx]

**Gene abbreviations in alphabetical order**

ACTB – Actin beta

AKT1 – RAC (Rho family)-alpha serine/threonine-protein kinase
ALIS –Aggresome-Like Induced Structures ATM – ATM Serine/Threonine Kinase
BCL2 – B-cell lymphoma 2
BRCA1 – Breast Cancer Type 1 Susceptibility Protein BIRC2 – Baculoviral IAP Repeat Containing 2 BIRC3 – Baculoviral IAP Repeat Containing 3
CD44 – CD44 molecule
CCNA1 – Cyclin A1
CCNA2 – Cyclin A2 CCNB1 – Cyclin B1 CCND1 – Cyclin D1 CDC42 – Cell Division Cycle 42

CDK1 – Cyclin-Dependent Kinase 1

CDK2 – Cyclin-Dependent Kinase 2

CDKN2A – Cyclin Dependent Kinase Inhibitor 2A

CHEK1 – Checkpoint Kinase 1

CTNNB1 – Catenin Beta 1

CYCS – Cytochrome C, Somatic

EGF – Epidermal Growth Factor

EGFR – Epidermal Growth Factor Receptor

EP300 – EP300 Lysine Acetyltransferase

ER – Endoplasmic Reticulum

ERBB2 – Erb-B2 Receptor Tyrosine Kinase 2

FCERI – High Affinity Immunoglobulin E Receptor

FN1 – Fibronectin 1

GAPDH – Glyceraldehyde-3-Phosphate Dehydrogenase

GRB2 – Growth factor receptor-bound protein 2

GSK3B – Glycogen Synthase Kinase 3 Beta

H3-3B – H3 Histone Family Member 3B

H4C6 – Histone H4 Clustered Protein 6

HDAC1 – Histone Deacetylase 1

HSPA5 – Heat Shock Protein Family A Member 5

HSP90AA1 – Heat Shock Protein 90 Alpha Family Class A Member 1

HSP90AB1 – Heat Shock Protein 90 Alpha Family Class B Member 1

IFNB 1 – Interferon beta 1

IL (1A, 1B/β, 6, 18) – Interleukin (1 alpha, 1 beta, 6, 18)

IKBKG – Inhibitor Of Nuclear Factor Kappa B Kinase Regulatory Subunit Gamma

JAK – Janus Kinase

JUN – Jun Proto-Oncogene

KRAS – KRAS Proto-Oncogene, GTPase

MAPK – Mitogen-Activated Protein Kinase MHC I – Major Histocompatibility Complex Class I MYC – MYC Proto-Oncogene NFKB1 – Nuclear factor kappa B (Subunit 1)

|  |
| --- |
| NFKBIA – NFKB inhibitor alpha  NOTCH – Neurogenic locus notch homolog protein  PLK1 – Polo Like Kinase 1  PKRN – PKRN Parkin RBR E3 Ubiquitin Protein Ligase  PIK3R1 – Phosphoinositide-3-Kinase Regulatory Subunit 1 |
| PTEN – Phosphatase and Tensin Homolog PTK2 – Protein Tyrosine Kinase 2 RHOA – Ras Homolog Family Member A RISC – RNA-Induced Silencing Complex RHO GTPase – Ras homolog gene family RPS27A – Ribosomal Protein S27a SLC39 (A1, A4, A6, A10) – Solute carrier family 39 members (A1, A4, A6, A10) SNCA – Synuclein alpha SRC – SRC Proto-Oncogene, Non-Receptor Tyrosine Kinase STAT3 – Signal transducer and activator of transcription 3 TNF – Tumor Necrosis Factor TP53 – Tumour protein 53 TRAF2 – TNF Receptor-Associated Factor 2 TRAF3 – TNF Receptor-Associated Factor 3 TRAF6 – TNF Receptor-Associated Factor 6 UBE2N – Ubiquitin Conjugating Enzyme E2 N UBA52 – Ubiquitin A-52 Residue Ribosomal Protein Fusion UBB – Polyubiquitin B UBC – Polyubiquitin C VCP – Valosin Containing Protein |
